# Left main coronary artery fistula to superior vena cava

**DOI:** 10.1259/bjrcr.20150387

**Published:** 2016-05-18

**Authors:** Yu Jin, Wei Li, Huaping Chen, Tao Liu, Zhiwei Guo, Guoqiang Xing, Sheng Zhang, QiWen Mu

**Affiliations:** ^1^ Sichuan Medical University, Chengdu, China; ^2^ Department of Radiology, Second Clinical Medical College of North Sichuan Medical College Nanchong Central Hospital, Nanchong, China; ^3^ Department of Cardiology, Second Clinical Medical College of North Sichuan Medical College Nanchong Central Hospital, Nanchong, China; ^4^ North Sichuan Medical University Nanchong Central Hospital, Nanchong, China; ^5^ Lotus Biotech.com LLC, John Hopkins University-MCC, Rockville, MD, USA; ^6^ Peking University Third Hospital, Bejing, China

## Abstract

Coronary artery fistula (CAF) is an uncommon vascular malformation. As the majority of patients remain asymptomatic, approximately half of the cases may be clinical undetectable. We report here a rare case of a 10-year-old female with CAF from the left main coronary artery to the superior vena cava detected on echocardiography and CT angiography.

## Summary

Coronary artery fistula (CAF) is defined as an abnormal communication that links one or more coronary arteries to the four chambers of the heart or the great vessels adjacent to the heart. CAF occurs in 0.002% of the general population and accounts for 0.4% for all cardiac malformations.^1^ Approximately half of the CAF patients are asymptomatic, and the fistula may spontaneously regress with age. Other CAF patients, however, could experience fatigue, atypical chest pain, exertional dyspnoea or other severe complications.^2,3^ The fistula commonly originates from the right coronary artery (RCA); the left coronary artery (LCA) is less frequently involved. In 40% of cases, the fistula drains into the right ventricle; in only 1% of cases does it drain into the superior vena cava (SVC).^4^ The rare CAF from the left main coronary artery (LMCA) to the SVC is reported in this study. Furthermore, a CT coronary angiogram (CTCA) illustrated aneurysmal dilatation of the LMCA and the SVC.

## Case presentation

A 10-year-old female was admitted to our department with a grade 3/6 continuous murmur on the right side of the chest. When she was 2 years old, a cardiac murmur was incidentally detected during a routine clinical examination. Echocardiography performed at the local hospital at that time showed CAF. However, the local doctor decided not to perform surgery considering her very young age and weak physical condition. During annual follow-up *via* transthoracic echocardiography, the fistula did not show spontaneous regression, although the patient was asymptomatic with a normal exercise capacity. On clinical examination, no other significant abnormality was noted.

**Figure 1.**
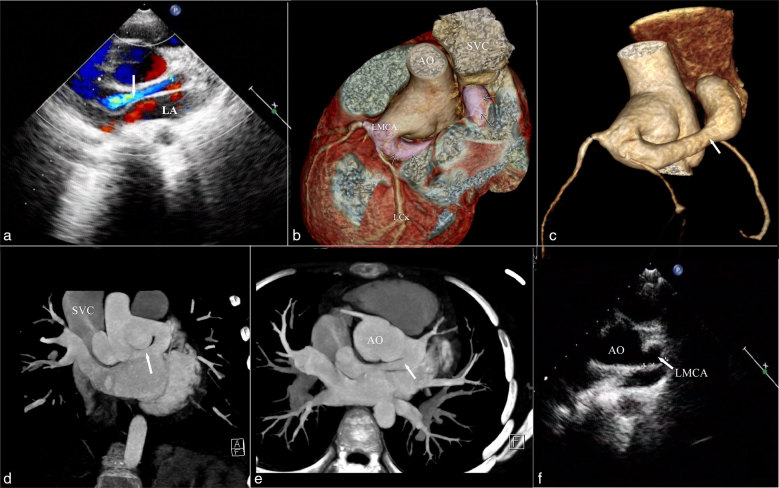
Pre-operative view (a–e). (a) Echocardiography showed a fistula in the parasternal long axis view. (b) DSCT with VR of the fistula, posterior view. (c) VR of coronary artery tree clearly demonstrating a connection between LMCA and SVC. (d) DSCT MIP, coronal view. (e) MIP, axial view. (f) Post-operative short-axis view of dilated LMCA (arrow indicates the closure of the fistula). Arrows in (a–e) indicate the fistula. AO, aorta; DSCT, dual-source CT; LA, left atrium; LMCA, left main coronary artery; MIP, maximum intensity projection; SVC, superior vena cava; VR, volume rendering.

A two-dimensional transthoracic echocardiography performed after admission revealed the abnormal vessel and the shunt rate of the fistula ostium (systolic period: 5.4 ms^–1^ and diastolic period: 3.5 ms^–1^), but normal ejection fraction (EF; 67%; Figure 1).

The CTCA (dual-source CT, SOMATOM Definition Flash; Siemens Medical Systems, Berlin, Germany) revealed particularly well the giant and tortuous vascular structure, located posterior to the root of the aorta and anterior to the left atrium (LA). The fistula had an overall length and width of over 7.7 and 2.0 cm, respectively; the maximum LMCA and SVC width were 1.3 and 4.2 cm, respectively. No apparent abnormality was seen at the origin and along the route of the left anterior descending, left circumflex and RCA (Figure 1b–e).

Following her doctor’s advice, the patient underwent on-pump surgical repair. During the operation, a fremitus of SVC could be felt and the fistula’s ostium in the SVC, approximately 4 mm, was sutured. 7 days later, an echocardiography was re-examined and showed no abnormal blood flow between the LMCA and the SVC (Figure 1f). The patient was discharged from the hospital in good condition.

## Discussion

CAF is an unusual but long-recognized cardiac anomaly. Its exact aetiology is unknown. To date, only two cases with CAF from LMCA to SVC have been reported in recent years.^5,6^

CAF is congenital in origin and mostly asymptomatic. Patients present symptoms often owing to progressive enlargement of the fistula and increasing left-to-right shunting,^3^ which causes ventricular overload and haemodynamic reduction of blood supply to the coronary artery. This shunting is commonly known as coronary steal phenomenon and should be diagnosed as early as possible owing to its asymptomatic nature and low morbidity.

Imaging techniques such as transthoracic echocardiography, transoesophageal echocardiography, CTCA, MRI and catheter angiography have allowed the depiction of a possible CAF; most of the previously mentioned procedures afford a better definition of CAF and can be helpful for diagnosis. However, some limitations are well recognized. Catheter angiography traditionally sets the gold standard.^7^ Transesophageal echocardiography is limited in that it best visualizes the LCA and the LA, and its use is considered somewhat invasive by some patients. Because of its non-invasive nature and no radiation exposure, transthoracic echocardiography is a better choice both before and after treatment to monitor and follow-up a CAF.^8^

CTCA and MRI should be performed once a diagnosis has been made; two- or three-dimensional reconstruction of CT scans could provide detailed images and better define the relationship of the fistula with the adjacent structures.^9^ Our current case highlighted the possibility of multiple non-invasive CT imaging modalities in the diagnosis of CAF. In addition to CTCA, MRI is also a good technique to discern the location, course, blood flow and function of the fistula, as it provides more information regarding tissue characteristics.^10,11^

The treatment strategies for patients with symptomatic CAF include surgery and transcatheterization, but to best treat the asymptomatic patients, the following factors are often taken into account when management options are made: the presence or absence of symptoms, the size and flow rate of the shunting fistula, complications and age.^12^ Taken together, when fistulas are diagnosed in young patients, surgical repairs prior to the development of more serious complications are thought to be the best choice.^13^

## Learning points

1. As CAF patients are asymptomtic, CAF should not be missed in our clinical work. CTCA or echocardiography is helpful for definitive diagnosis and CTCA is often required to obtain sufficient information prior to making an appropriate therapeutic decision.
2. CTCA or echocardiography as non-invasive examinations could be used to clarify the site (its origin and termination), length, width and complex malformation of a CAF.

## Consent

The study was approved by, and in compliance with the ethical standards of the North Sichuan Medical University Institutional Review Board. Informed consent was obtained from the patient's parents prior to the study.
